# Metagenome-assembled genomes from sugarcane mill mud

**DOI:** 10.1128/MRA.00568-23

**Published:** 2023-10-17

**Authors:** Minori Uchimiya, Leah E. Elliott, Christopher M. Derito, Anthony G. Hay

**Affiliations:** 1 United States Department of Agriculture, Agricultural Research Service, Southern Regional Research Center, New Orleans, Louisiana, USA; 2 Department of Biological and Chemical Sciences, Wells College, Aurora, New York, USA; 3 Department of Microbiology, Cornell University, Ithaca, New York, USA; Portland State University, Portland, Oregon, USA

**Keywords:** biofertilizers, soil microbiology, biogeochemistry

## Abstract

The genomes of 11 bacteria and 3 archaea were assembled from metagenomic DNA extracted from sugarcane mill mud. These metagenome-assembled genomes ranged from 1.79 to 6.45 Mb, with 2,263 to 5,551 predicted proteins, 80.65% to 100% genome completeness, and 43.19% to 68.02% G+C content.

## ANNOUNCEMENT

Sugarcane mill mud is a biomass byproduct formed during raw sugar production through a heated sedimentation process. It has garnered interest as a biofertilizer and biostimulant because of its nutrient and microbial contents (1). We recently described the bacterial 16S rRNA gene sequences for the sample analyzed in this report along with several other mill muds (1). This sample came from a dense slurry formed by historical on-site disposal of mill mud [“Mud-LA1-O” described in detail in reference (1)].

DNA was extracted from mill mud using E.Z.N.A Soil DNA Kit (OMEGA Bio-Tek, Doraville, USA) as described previously (1). The metagenomic library was prepared using a Nextera XT Kit (Illumina, San Diego CA, USA) and sequenced on an Illumina NextSeq2K using NextSeq P3 300 bp (2 × 150), producing 154,651,792,150 bp paired-end reads.

Raw sequence reads were uploaded to KBASE and processed there as described below (2). Unless otherwise noted, default parameters were used on all software. Reads with an average quality of ≤15 were filtered out, and sequencing adaptors were removed using Trimmomatic v0.36 (3). Quality was ascertained by Quast v4.4 (4). The reads were assembled *de novo* using Megahit v1.2.9 (5) generating 148,538 contigs >1 Kb, 60% of which were binned using MaxBin v2.2.4 (6). These were extracted with BinnedUtil v1.0.2 (2). CheckM v1.0.18 (7) was used to estimate completeness, heterogeneity, and contamination. The metagenome-assembled genomes (MAGs) were annotated using the NCBI Prokaryotic Genome Annotation Pipeline (8 – 10). Taxonomies were assigned using GTDB-Tk v1.7.0 (11) and its dependencies (12 – 20). The percentage of metagenomic reads that each taxonomic group represented was determined with Kaiju (21).

We assembled 14 MAGs with >75% completeness and <10.6% contamination (Table 1). The MAGs include representatives of 10 phyla from the domains Bacteria and Archaea: all were unique at the species level or above. The closest taxonomic match was phylum for 4, class for 6, order for 7, and family for 10 of the MAGs. All the archaeal genomes were in the family Methanotrichaceae. Overall, archaea represented 5% of the total metagenomic reads. The bacterial MAGs represented eight phyla; half of which (Acidobacteriota, Bacteroidota, Chloroflexota, and Planctomycetota) have previously been reported in mill mud (1). These eight bacterial phyla represented ~40% of total metagenomic reads.

**TABLE 1:** Assembly characteristics, name, and taxonomic rank of closest relative, and GenBank accession number for bacterial and archaeal MAG

MAG name	GTDB-TK taxonomy^*a*^	NCBI taxonomy	Completeness (%)	Contamination (%)	Heterogeneity (2+)	GC (%)	Annotation size (bp)	No. of contigs	N50	No. of putative proteins	Accession number
Bin.004	d *Bacteria*; p *Zixibacteria*; c MSB-5A5; o GN15; f FEB-12; g__; s__	Candidate division *Zixibacteria bacterium*	100	2.2	1.4	63.39	3,542,859	179	4,416	3,100	JAUCPV000000000
Bin.010	d *Bacteria*; p *Fermentibacterota*; c *Fermentibacteria*; o *Fermentibacterales*; f *Fermentibacteraceae*; g *Fermentibacter*; s__	*Candidatus Fermentibacter* sp.	91.76	1.28	1.4	66.34	2,549,338	72	77,071	2,464	JAUCPW000000000
Bin.011	d *Bacteria*; p *Desulfobacterota*; c *Syntrophia*; o *Syntrophales*; f *Smithellaceae*; g *Smithella*; s__	*Smithella* sp.	96.77	6.15	4.5	46.22	3,451,210	125	64,279	3,450	JAUCPX000000000
Bin.015	d *Bacteria*; p *Bacteroidota*; c *Bacteroidia*; o *Bacteroidales*; f VadinHA17; g SR-FBR-E99; s__	*Bacteroidota bacterium*	95.87	9.23	7.3	51.19	3,891,448	546	14,479	3,483	JAUCPY000000000
Bin.016	d *Bacteria*; p *Chloroflexota*; c *Dehalococcoidia*; o UBA2777; f RBG-13–53-26; g__; s__	*Dehalococcoidia bacterium*	95.71	8.14	7.9	59.53	3,151,809	349	19,275	3,246	JAUCPZ000000000
Bin.017	d *Bacteria*; p *Acidobacteriota*; c UBA6911; o UBA6911; f UBA6911; g JAAYAM01; s__	*Acidobacteriota bacterium*	95.94	9.4	6.4	55.38	5,244,046	296	33,341	5,021	JAUCQA000000000
Bin.018	d *Bacteria*; p *Planctomycetota*; c *Phycisphaerae*; o UBA1845; f PWPN01; g JAAXZI01; s__	*Phycisphaerae bacterium*	97.73	5.32	4.9	62.43	6,453,651	272	59,607	5,551	JAUCQB000000000
Bin.019	d *Bacteria*; p QNDG01; c QNDG01; o QNDG01; f__; g__; s__	*bacterium*	84.62	6.59	4.1	63.14	3,283,253	304	20,949	2,924	JAUCQC000000000
Bin.020	d *Archaea*; p *Halobacteriota*; c *Methanosarcinia*; o *Methanotrichales*; f *Methanotrichacea*e; g *Methanothrix*; s__	*Methanothrix* sp.	97.22	1.96	1.3	51.98	2,111,874	291	11,328	2,486	JAUCQD000000000
Bin.028	d *Bacteria*; p *Acidobacteriota*; c *Blastocatellia*; o *Pyrinomonadales*; f *Pyrinomonadaceae*; g OLB17; s__	*Pyrinomonadaceae bacterium*	95.44	6.67	6.4	56.12	3,810,823	718	8,403	4,066	JAUCQE000000000
Bin.038	d *Archaea*; p *Halobacteriota*; c *Methanosarcinia*; o *Methanosarcinales*; f *Methanosarcinaceae*; g *Methanosarcina*; s__	*Methanosarcina* sp.	85.4	9.52	11.0	43.19	3,151,424	1,029	4,351	3,867	JAUCQF000000000
Bin.044	d *Bacteria*; p *Eisenbacteria*; c RBG-16–71-46; o CAIMUX01; f__; g__; s__	*Candidatus Eisenbacteria bacterium*	81.42	10.54	7.5	68.02	3,903,488	1,564	2,936	2,460	JAUCQG000000000
Bin.045	d *Archaea*; p *Halobacteriota*; c *Methanosarcinia*; o *Methanotrichales*; f *Methanotrichaceae*; g *Methanothrix*; s__	*Methanothrix* sp.	96.16	9.35	7.9	58.29	1,787,686	566	4,135	2,263	JAUCQH000000000
Bin.063	d *Archaea*; p *Halobacteriota*; c *Methanosarcinia*; o *Methanotrichales*; f *Methanotrichaceae*; g JABLXE01; s__	*Methanotrichaceae archaeon*	80.65	5.58	4.4	53.69	1,826,065	831	2,469	2,460	JAUCQI000000000

^*a*^ p, phylum; c, class; o, order; f, family; g, genus; s, species.

Arguably, the most interesting of the MAGs hails from the poorly characterized *Zixibacteria* candidate division in the Fibrobacterota, Chlorobiota, and Bacteroidota (FCB) group (22). Discovery of this MAG in mill mud increases the known range of *Zixibacteria* which had previously been reported in sediments, soils, and duck guts (23 – 27). Our *Zixibacteria* MAG is predicted to encode genes for diverse carbohydrate metabolism, fermentation, anaerobic respiration, superoxide dismutase, catalase, and resistance to arsenic, zinc, cobalt, and copper. It is also predicted to encode genes for flagellar motility.

## Data Availability

The raw metagenomic reads (SRR25629172) and MAGs were deposited at GenBank under the BioProject accession number PRJNA911693. Individual accession numbers for each MAG are listed in Table 1.
